# Population Stratification of a Common *APOBEC* Gene Deletion Polymorphism

**DOI:** 10.1371/journal.pgen.0030063

**Published:** 2007-04-20

**Authors:** Jeffrey M Kidd, Tera L Newman, Eray Tuzun, Rajinder Kaul, Evan E Eichler

**Affiliations:** 1 Department of Genome Sciences, University of Washington, Seattle, Washington, United States of America; 2 Howard Hughes Medical Institute, University of Washington, Seattle, Washington, United States of America; University of Oxford, United Kingdom

## Abstract

The *APOBEC3* gene family plays a role in innate cellular immunity inhibiting retroviral infection, hepatitis B virus propagation, and the retrotransposition of endogenous elements. We present a detailed sequence and population genetic analysis of a 29.5**-**kb common human deletion polymorphism that removes the *APOBEC3B* gene. We developed a PCR-based genotyping assay, characterized 1,277 human diversity samples, and found that the frequency of the deletion allele varies significantly among major continental groups (global *F*_ST_ = 0.2843). The deletion is rare in Africans and Europeans (frequency of 0.9% and 6%), more common in East Asians and Amerindians (36.9% and 57.7%), and almost fixed in Oceanic populations (92.9%). Despite a worldwide frequency of 22.5%, analysis of data from the International HapMap Project reveals that no single existing tag single nucleotide polymorphism may serve as a surrogate for the deletion variant, emphasizing that without careful analysis its phenotypic impact may be overlooked in association studies. Application of haplotype-based tests for selection revealed potential pitfalls in the direct application of existing methods to the analysis of genomic structural variation. These data emphasize the importance of directly genotyping structural variation in association studies and of accurately resolving variant breakpoints before proceeding with more detailed population-genetic analysis.

## Author Summary

Several recent studies have demonstrated that deletions, duplications, and inversions contribute a substantial fraction of the total amount of variation present in the human genome. In this study, we provide a comprehensive population-genetic analysis of a single deletion previously identified by comparing the genome of a single individual against the human genome reference sequence. Complete genomic sequence spanning the deleted region was obtained, allowing us to define the deletion breakpoints and develop a direct genotyping assay. Analysis showed that the deletion removes a member of a gene family involved in the innate immune response against viral pathogens. We genotyped samples from a human diversity panel and found drastic differences in the frequency of the deletion around the world. Using data from the HapMap project and the application of existing analysis techniques, we illustrate the importance of directly genotyping this type of variation and of clearly defining its boundaries. Without this level of detail the potential functional importance of such variation may be missed.

## Introduction

The *APOBEC3* family is known to play a role in innate cellular immunity against retroviral infection. The gene family has undergone an expansion in primates, increasing from a single copy in rodents to at least seven copies in humans [1–3]. Among primates, the *APOBEC3* family has been subjected to strong and continuing selective pressures at the amino acid level [3,4]. APOBEC3 proteins defend against retroviruses by deaminating cytosine residues to uracil, resulting in hypermutation and degradation of the viral genome. Members of this gene family contain either one *(APOBEC3A* and *APOBEC3C)* or two *(APOBEC3B, APOBEC3F,* and *APOBEC3G)* conserved cytosine deamination domains [1,2]. In addition to their role in innate retroviral immunity, some *APOBEC3* genes appear to inhibit hepatitis B virus infection [5–8] and the retrotransposition of endogenous elements [9–12]. It is thought that at least part of this activity occurs through a deamination-independent mechanism [12].

Several recent studies have brought increased attention to classes of genomic variation such as deletions, inversions, and copy-number polymorphisms [13–20]. It is thought that these variations contribute substantially to inter-individual genomic, and perhaps, phenotypic variation, but the structure and population characteristics of these variants remain largely unexplored. A deletion in the *APOBEC3* gene cluster was recently identified using two different approaches. The deletion was first discovered by mapping end-sequence pairs from a human fosmid library against the human genome reference sequence assembly [16]. A cluster of discordant fosmid clones whose end-sequences mapped further apart than the expected fosmid insert size predicted a deletion of ∼30 kb near the *APOBEC3B* gene. Later, a second approach confirmed the deletion based on an interrogation of a dense single nucleotide polymorphism (SNP) marker map generated as part of the International HapMap Project [17]. This method discovered deletions by identifying clusters of SNPs that showed apparent non-Mendelian inheritance, deviations from Hardy-Weinberg equilibrium, or evidence of null genotypes. Nevertheless, this variant was not detected in a recent genome-wide screen of structural variation in the HapMap populations using BAC or SNP-based microarrays [20]. The availability of a fosmid clone that captured the deletion event allowed us to sequence the structural variant in its entirety and confirm its presence. Precise sequence definition of the deletion enabled the design of specific genotyping assays across the deletion breakpoints. We present here a sequence-based analysis of this deletion polymorphism, a worldwide population survey of the deletion frequency (1,277 DNA samples), and an analysis of the surrounding haplotype structure. The results suggest this is a functionally important structural variant that is stratified in the human population.

## Results

### Sequence-Based Resolution of Deletion Breakpoints

**Figure 1:**
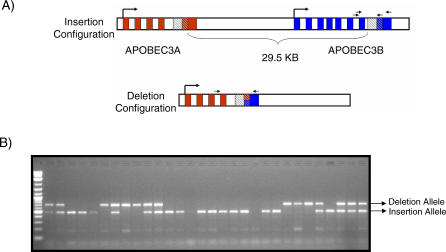
Sequence Structure of Structural Variant and Genotyping Assay Design (A) A fosmid clone containing the deletion variant was completely sequenced and compared to the human reference genome (insertion allele). The deletion removes a 29.5-kb region spanning from the fifth exon of *APOBEC3A* (shown in red) to the eighth exon of *APOBEC3B* (shown in blue). The deletion breakpoints map within identical 350-bp blocks (hatched shading) and the deletion is predicted to form a hybrid transcript. PCR assays were designed to distinguish insertion and deletion alleles (short arrows show position of PCR oligonucleotides). (B) PCR breakpoint genotyping assay. Genotyping results for 30 individuals are shown (lane 20 = negative water control lane). All homozygous deletion events were confirmed by a second PCR assay designed to the insertion breakpoint (see Materials and Methods).

We sequenced the entire insert of one of the fosmid clones whose end-sequence pairs had initially identified the structural variant. Alignment of this sequence with the sequence from the finishing human genome sequence assembly (hg17) confirmed the presence of a deletion overlapping the *APOBEC3A* and *APOBEC3B* transcripts (Figure 1). Consistent with non-allelic homologous recombination as the likely mechanism of origin, the deletion breakpoints mapped to two highly identical tracts of sequence: 350 bp in length, 100% sequence identity. In the deleted configuration, a single copy of this sequence exists and the 29.5 kb of sequence between them is removed (position 37,683,131–137,712,716 on Chromosome 22 of hg17). The deletion removes the genomic sequence between the fifth exon of *APOBEC3A* and the eighth exon of *APOBEC3B,* leading to a predicted full-length functional hybrid transcript with a predicted amino acid composition identical to *APOBEC3A.* Thus, individuals possessing this structural variant would lack at least one copy of the unique coding portion of *APOBEC3B.* Interestingly, the predicted transcript would contain the 3′ UTR from *APOBEC3B,* but be subject to *APOBEC3A* upstream regulatory signals.

### Deletion Frequency

**Figure 2:**
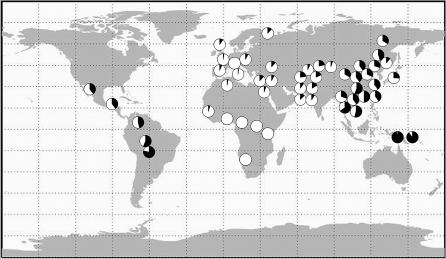
Worldwide Frequency Distribution of *APOBEC3B* Deletion Genotype frequencies from analysis of the HGDP (51 populations, Table S3) are overlaid on a map of the world. The deletion (black area of pie chart) and insertion frequency (white) are depicted.

The availability of the complete sequence of the deletion breakpoints allowed us to design PCR breakpoint assays which distinguished insertion and deletion alleles (Figure 1 and Materials and Methods). We genotyped 1,007 individuals from 51 populations included in the Centre d'Etude du Polymorphisme Humain (CEPH) Human Genome Diversity Panel (HGDP) and found that the deletion frequency was highly variable (Figure 2). The deletion is rare in African and European populations (frequency of 0.9% and 6%, respectively), more common in East Asian and American populations (36.9% and 57.7%), and almost fixed in Oceanic populations (92.9%; Tables S1–S3). As a control against potential SNPs under the PCR primer binding sites leading to an overestimate of the frequency of homozygotes, we reanalyzed all 127 samples initially scored as deletion homozygotes with a second PCR assay targeted to the insertion allele (see Materials and Methods). We reclassified 25 samples (19.6%) as hemizygous (Table 1). In order to rule out further large-scale genotyping error, we calculated estimates of Hardy-Weinberg equilibrium for each population. No significant deviations were observed.

**Table 1:**
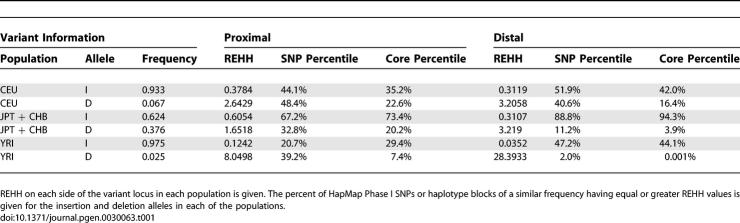
REHH

### *F*_ST_ Analysis

As a measure of population differentiation, we calculated an overall *F*_ST_ value of 0.2843 for the *APOBEC3B* deletion in the HGDP. Large *F*_ST_ values are consistent with either geographically restricted selection (local adaptation) or demographic history (i.e., population bottlenecks and founder effects). To distinguish between these two possibilities, we calculated an empirical *F*_ST_ distribution using 2,540 autosomal SNPs and 207 small indels genotyped in individuals from the same 51 populations [21,22]. Of the 2,747 loci, 52 had an *F*_ST_ value greater than that obtained for the *APOBEC3B* deletion, placing this deletion within the top 2% of the empirical distribution. Estimates of *F*_ST_ may be sensitive to allele frequency, so we repeated this comparison by only considering the 635 loci that had a global minor allele frequency between 0.17 and 0.27. A single SNP (rs2250341, located in an intron of the *PCP4* gene) of this subset had an *F*_ST_ value greater than the *APOBEC3B* deletion, placing the deletion in the top 0.16% of the frequency-matched empirical distribution.

A striking feature of the deletion is the clinal increase in frequency as one moves eastward away from Africa (Figure S1). In order to further delineate this pattern, we repeated this analysis using pairwise *F*_ST_ estimates between all possible combinations of the 51 populations (Figure S2). The analysis differentiates Oceanic, Amerindian, and some East Asian populations from other human populations based on the frequency of the deletion variant in comparison to other SNP and indel loci in the same populations. This suggests that this pattern is not solely the result of demographic history.

### Linkage Disequilibrium and Haplotype Structure

**Figure 3:**
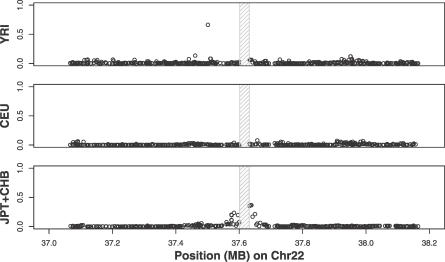
LD Flanking the Deletion Variant LD (*r* ^2^) between the deletion locus (shaded box) and flanking Phase I HapMap SNPs is depicted for the YRI, CEU, and JPT + CHB populations.

We genotyped the individuals included in the HapMap project: consisting of samples from the Yoruba people of the Ibidan Peninsula in Nigeria (referred to as YRI), the CEPH project in Utah (CEU), the Han Chinese population of Beijing (CHB), and individuals of Japanese ancestry from the Tokyo area (JPT); and searched for evidence of linkage disequilibrium (LD) between the *APOBEC3B* deletion and flanking HapMap Phase I SNPs (Tables S4–S5). In contrast to other deletion polymorphisms [17,19], we found no single SNP to be in strong LD (*r* ^2^ greater than 0.8) with the deletion variant (Figure 3). In the Yoruba sample there was one rare SNP with an *r* ^2^ value of 0.663. This SNP, rs733107, has a minor allele frequency of 0.025 with two of the three Yoruba deletion chromosomes carrying the minor allele. Interestingly, we also found that no two-marker combination had an *r* ^2^ value greater than 0.8 (the maximum values are 0.254 for CEU, 0.499 for JPT and CHB, and 0.661 for YRI). However, it remains possible that more sophisticated multi-marker approaches will be able to successfully tag the deletion variant using existing SNPs. Unlike other regions of the human genome, such as those enriched for complex segmental duplications, the SNP density in this region (approximately one SNP every 2.5 kb) is not significantly reduced. Since the deletion frequency shows drastic differences among the HapMap populations, the absence of a suitable single marker tag is likely a consequence of a bias in the ascertainment of SNPs typed in the HapMap panel.

**Figure 4:**
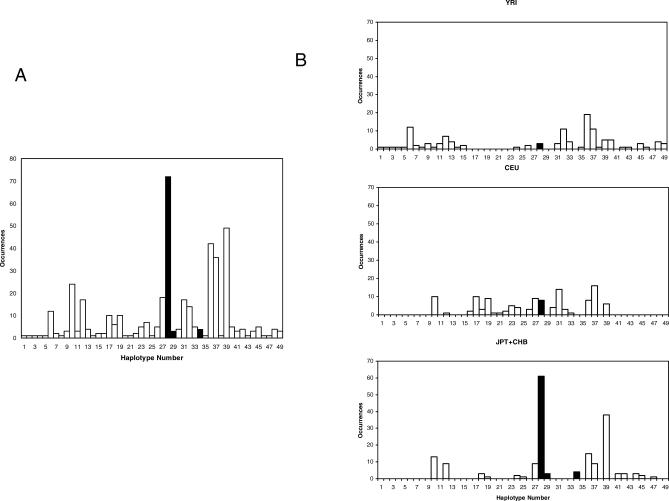
*APOBEC3B* Haplotype Structure The deletion locus was coded as a bi-allelic variant, and phased haplotypes were constructed for the locus and all Phase II SNPs within 25 kb of the deletion. The black bars identify haplotypes that carry the deletion allele while the white bars carry the insertion allele. The number of occurrences for each haplotype is shown for all of the HapMap samples (A) and for each population separately (B).

Although no single SNP can act as a reliable proxy for the variant, we noticed that the deletion appears to occur on a common haplotype. Treating the deletion locus as a bi-allelic variant, we constructed phased haplotypes using 21 HapMap Phase II SNPs genotyped in all populations and located within 25 kb of the deletion boundaries and performed a haplotype network analysis (Figures 4 and S3; Tables S6 and S7) [23] (http://fluxus-engineering.com).

We identified 49 distinct haplotypes over this region. Overall, 91% of the deletion events (YRI = 3/3, CEU = 8/8, and 61/68 of the JPT and CHB) lie on a single common haplotype (haplotype 28) which differs from haplotype 31 only by the presence of the deletion variant. Two additional haplotypes are observed only for JPT and CHB deletion chromosomes: haplotype 34 (*n* = 4 chromosomes) and haplotype 29 (*n* = 3 chromosomes) differ from haplotype 28 by a single nucleotide difference. The former may represent an independent occurrence of the deletion on haplotype 39 or a recombination event between haplotypes 39 and 29.

### Extended Haplotype Homozygosity

**Figure 5:**
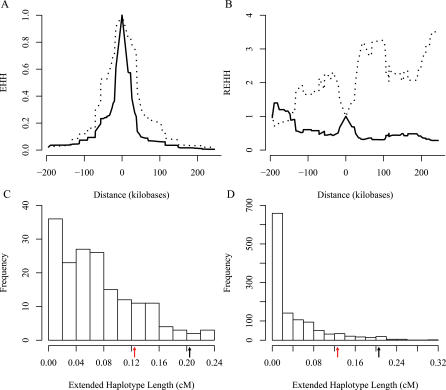
Impact of Deletion Size on EHH (A) EHH and (B) REHH values are shown for the insertion allele (solid line) and deletion allele (dotted line) in the combined JPT and CHB HapMap population. Distances are plotted based on the given SNP coordinates without accounting for the reduced size of the deletion haplotype. The extended haplotype length for the deletion variant is compared to an empirical distribution obtained for (C) SNP haplotype blocks and (D) individual SNPs. The comparison was made with SNPs or cores having a frequency between 0.35–0.40. The black arrow indicates the value obtained without correcting for the reduced size of the deletion haplotype while the red arrow indicates the true EHL value for the deletion.

In order to further investigate the unusual shared haplotype structure and potential signatures of selection, we assessed the deletion haplotype for evidence of extended homozygosity [24,25]. We calculated extended haplotype homozygosity (EHH), the relative extended haplotype homozygosity (REHH), and the extended haplotype length (EHL) for both the deletion and insertion alleles (Figures 5 and S4; Tables 1 and 2). Without correcting for the decreased size of the deletion haplotype, a potentially strong signal of local adaptation indicated by a high frequency extended haplotype for the deletion allele in Asia is observed (Figure 5B and 5C; Table 3). Accounting for the physical reduction in chromosome size due to the deletion, however, largely eliminates this signal in the Asian population (Table 2). Nevertheless, the haplotype analysis does suggest weak signals of selection, particularly in the Yoruba population.

**Table 2:**
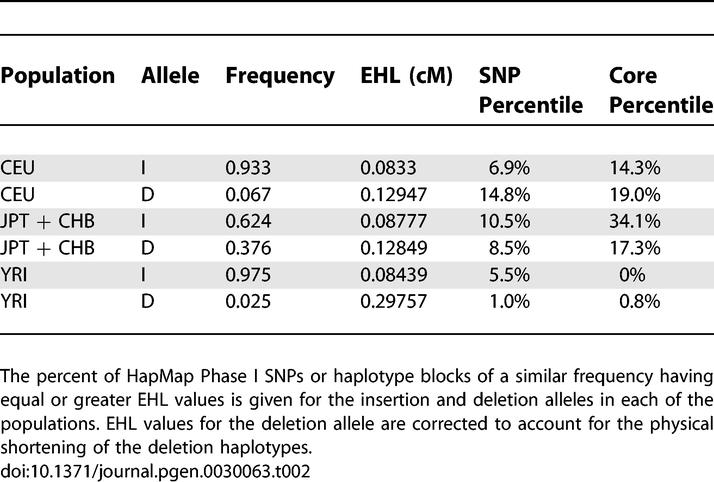
EHL and the Impact of Shortened Deletion Haplotypes

**Table 3:**
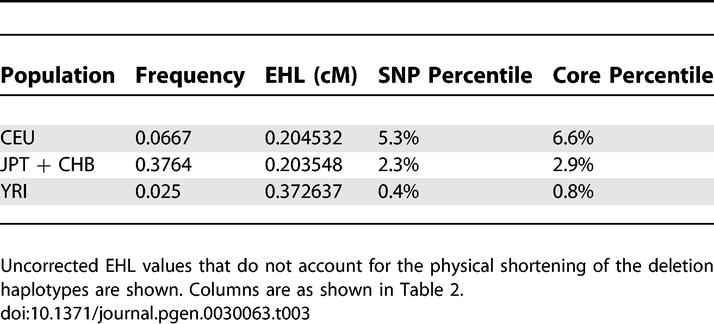
Uncorrected EHL Values for Deletion Haplotypes

## Discussion

There are three important results of this examination of the *APOBEC3B* deletion. First, the deletion occurs between two asymmetric gene structures *(APOBEC3B* and *3A)* and produces a hybrid transcript whose putative coding sequence maintains its frame. Despite the fact that the recombination event occurs between coding exons, the amino acid sequence of the hybrid gene is identical to *APOBEC3A* with the net effect being complete loss of *APOBEC3B* and potential altered regulation of *APOBEC3A* due to juxtaposition of novel 3′ regulatory sequences. Second, the deletion variant shows dramatic population stratification with significantly elevated *F*_ST_ values observed for Eastern Asian, Oceanic, and Amerindian populations. The magnitude of the *F*_ST_ values compared to a set of other genome-wide loci from the same populations suggest that these observed frequency differences are not due to demographic history alone. Third, we observe that no tag-SNP currently exists for this deletion. A sophisticated, multi-marker tagging approach may successfully tag this allele, but this approach is complicated by the observation that the major deletion haplotype (haplotype 28) is identical to another haplotype (haplotype 31), except for the presence of the insertion allele. Thus, despite the fact that nearly 40% of the world's population carries at least one copy of this deletion, a suitable SNP surrogate does not yet exist. In light of its abundance, it is noteworthy that this variant was not detected in a recent genome-wide screen of copy-number variation in the human genome [20].

These data emphasize that the phenotypic impact of this and potentially other structural variants may be overlooked in association studies unless the structural variant is directly genotyped. We also observed that potentially misleading results can be obtained from the direct application of existing haplotype-based methods for detecting selection to structural variants. In our study, adjusting for the length of the haplotype reduced the significance of signals of selection using EHL methods (Figure 5). This highlights the importance of not only directly genotyping this type of variation, but of also clearly resolving the breakpoints of the event. If genome-wide screens for selection based on EHL are performed without controlling for changes in the length of the genome sequence, artifactual signals may be observed.

Our observations of the deletion architecture, the population frequency patterns, and the haplotype structure indicate that variation at this locus may be important. Analysis also revealed a weak, suggestive signal of selection for the deletion. Since it appears that the deletion occurred once and has since spread into other populations, a complete understanding of the history of this locus would require detailed knowledge of the different selective regimes and demographic histories unique to these populations.

Until a more comprehensive population genetic theory is developed and a phenotypic consequence of the deletion event is demonstrated, one must be cautious about placing too much emphasis on a potential selective advantage for the deletion. Nonetheless, several possible scenarios may account for the patterns observed at this locus. First, it is possible that a 29.5-kb deletion of a gene may have altered the properties of genetic recombination on this specific haplotype. Long-range haplotype tests are highly sensitive to variance in recombination rates among different haplotypes [25]. Suppressed recombination could, in principle, retard LD decay resulting in an underestimate of the allele's age leading to erroneous signals of recent selection. If this were the case, one would expect a striking correspondence between long-range haplotype-based signatures of selection [26] and the more than 1,000 deletion polymorphisms that have now been documented for the human genome [13–20]. Such a correspondence has not yet been observed, although few structural variants have been studied at this level of detail.

In a second scenario, the deletion may simply be a genetic marker for another selective event that has occurred on this specific haplotype (haplotype 28). In this model, the deletion event is a genetic hitchhiker as opposed to the causative allele. The *APOBEC3* gene cluster has been subject to positive selection at many different time points during human and primate evolution [3,4,26]. Thus, a partial sweep of the deletion variant could occur if individuals carrying this specific haplotype had greater resistance to specific pathogens, perhaps as a result of amino acid changes in adjacent members of the *APOBEC* gene family. In such a scenario, however, the fitness advantage would have to outweigh loss of the *APOBEC3B* gene and the potential regulatory changes of *APOBEC3A* incurred by the disruption to the surrounding genomic region. A similar scenario has been put forward for rearrangements associated with the alpha-globin gene family. In this case, recurrent deletions of Hba1 and Hba2 associated with alpha-thalassemia have risen to high frequency in Mediterranean and Pacific rim populations [27]. Data from Papua New Guinea suggest that homozygous and heterozygous genotypes confer protection against malaria and other infectious disease [28]. A neutral deletion seems unlikely in light of the conservation of this gene in humans and other great ape species ([29] and J. M. Kidd, unpublished data). Moreover, *APOBEC3B* has recently been shown to inhibit replication of the hepatitis B virus, an observation that may warrant further study in light of the frequency of this deletion [8,30].

In a third scenario, the increased frequency of the deletion could represent a shift in the balance of selective forces impacting this locus. There may be a significant cost associated with the maintenance of active cytidine deaminases due to their mutagenic potential [3,31,32]. It has been shown that APOBEC3B protein is present in the nucleus of cells where it may act to repress retrotransposition in early stages of development and in the germ line [8,9]. When the threat posed by both endogenous and exogenous viral activity is high, the protective properties offered by *APOBEC3B* may outweigh the risks associated with its own activity. When rates of endogenous viral activity are low and when changes in environment reduce the presence of exogenous virus activity, the detrimental effects associated with *APOBEC3B* may outweigh its benefit, resulting in strong selective pressure in favor of the deletion. Similar arguments have recently been proposed to account for the presence of an impaired allele of the retroviral defense gene *TRIM5α* and is consistent with evidence of increased retroviral activity in African but not Asian apes [33,34]. When it comes to innate immune system genes such as *APOBEC3B,* less truly may be more [35].

## Materials and Methods

### Sequencing and sequence analysis.

A shotgun sequence assembly of the fosmid insert containing the putative deletion was generated as previously described [16]. Prior to sequencing, a fingerprint map with four independent restriction enzymes (EcoRI, HindIII, BglII, and NsiI) confirmed the ∼30-kb deletion [36]. Deletion breakpoints were identified by comparison of the fosmid insert sequence against the human genome reference sequence assembly (hg17) using ClustalW and BLASTN [37,38].

### PCR genotyping assay.

We designed PCR breakpoint assays to distinguish the insertion and deletion alleles based on the following oligonucleotide sequences: Deletion_F: TAGGTGCCACCCCGAT; Deletion_R: TTGAGCATAATCTTACTCTTGTAC; Insertion1_F: TTGGTGCTGCCCCCTC; Insertion1_R: TAGAGACTGAGGCCCAT; and Insertion2_F: TGTCCCTTTTCAGAGTTTGAGTA; Insertion2_R: TGGAGCCAATTAATCACTTCAT. Deletion primers are specific to the deletion sequence configuration and generate a 700-bp PCR product upon amplification. Insertion1 and Insertion2 primers amplify only the insertion configuration and produce 490- and 705-bp products, respectively. Insertion and deletion PCR assays were performed separately, the products pooled, and visualized on a standard 1.5% agarose gel. PCR was performed in 17-μl reactions composed of 0.85 μl of a 10-μM dilution of the forward primer, 0.85 μl of a 10-μM dilution of the reverse primer, 8.5 μl of Qiagen (http://www1.qiagen.com) PCR mastermix, and 50 ng of DNA. The following cycling conditions were used: 5 min at 95 °C, followed by 40 cycles at 95 °C for 1 min, 60 °C for 1 min, and 72 °C for 1 min, followed by 7 min at 72 °C. Each individual from the HapMap was genotyped in replicate with the Deletion and Insertion1 primers while each individual in the HGDP was genotyped one time. In addition, each of the samples, which appeared to be homozygous for the deletion, were genotyped using a second set of oligonucleotides for the insertion (Insertion2)

### Human DNA samples.

We genotyped 1,277 DNA samples corresponding to 270 samples from the International Hap Map project and 1,007 individuals from the CEPH HDGP [39,40]. Our analysis from the HGDP includes individuals from 51 different populations and excludes samples previously identified as duplicates [21,41,42]. Eight individuals (numbers 993, 994, 1028, 1030, 1031, 1033, 1034, and 1035) belonging to South African Bantu populations were genotyped (each was homozygous for the insertion) but were not included in the analysis due to small sample size. Individual genotypes are provided in Tables S1 and S4.

### Haplotype construction.

Phased SNP genotypes from HapMap Phase I were used for LD and extended haplotype analyses (http://hapmap.org). We excluded SNPs mapping within the deleted region and used PHASE version 2.1 to infer new haplotypes which included the insertion/deletion genotypes (http://www.stat.washington.edu/stephens/software.html) [43,44]. We also constructed haplotypes for insertion/deletion alleles based on the more complete Phase II genotyping data using only SNPs genotyped in all populations (Table S6).

### Population genetic analysis.

We used an exact test of Hardy-Weinberg equilibrium for two-allele loci as implemented in version 1.2.0 of the R genetics package [45]. LD was measured by *r* ^2^. *F*_ST_ values were calculated from population allele frequencies using an unbiased estimator [46,47]. The calculated *F*_ST_ values were compared with an empirical distribution defined by a collection of SNPs and small indels genotyped in the same individuals [21,22].

EHH, REHH, and EHL were calculated for the locus using SWEEP (http://www.broad.mit.edu/mpg/sweep/index.html) version 1.0 [24]. EHH is the probability that two randomly chosen chromosomes carrying the same allele at a core region are homozygous for all SNPs to a defined distance (x) from the core. REHH measures the decay of EHH at a given core genotype compared to the decay of other core haplotypes. SNPs mapping within the deleted region (five SNPs in CEU and two SNPs each in JPT and CHB and YRI) were excluded for all analyses of both the insertion and deletion cores. We measured REHH values at a marker H of 0.04, which is a measure of the observed amount of recombination and is roughly equal to a genetic distance of 0.25 cM. Observed REHH values on each side of the core were compared with REHH values calculated for each HapMap Phase I SNP. EHL is operationally defined as the sum of the genetic distance at which EHH falls to 0.5 on either side of the core [48]. This distance is sensitive to the density of markers, so SNP density was controlled by matching to the density around *APOBEC3* using SWEEP (approximately one SNP every 2,500 bases). In this analysis, the core haplotype was defined as simply the insertion or deletion genotype. Comparisons were made with an empirical distribution calculated from HapMap Phase I data using two different definitions for the core region. First, we defined the core as the longest non-overlapping haplotypes containing between three and ten SNPs [49]. Secondly, we treated each SNP locus individually as a core. Resulting values were then divided into 20 bins based on the core frequency (intervals of 5%), and the values corresponding to the insertion and deletion alleles for each population were compared.

For the EHL analysis, haplotype length was measured in two ways. For the insertion, the core was placed at the center of the variant region, and EHL was calculated as the sum of the genetic distance at which EHH fell to 0.5 on the proximal and the distal sides of the core. The application of the same procedure to the deletion core results in a misleading haplotype length since the corresponding haplotype on deletion chromosomes is physically shorter due to the deletion. In order to account for this, the haplotype length in the proximal and distal directions was calculated separately by defining each breakpoint as the position of the core. This assures that the length of the extended haplotype is not inflated by the inclusion of the chromosomal segment which is actually deleted.

## Supporting Information

Figure S1 Clinal Pattern of Allelic Variation Deletion frequency is plotted against longitude to illustrate the increased prevalence of the deletion moving eastward away from Africa.
(60 KB PPT)

Figure S2 Pairwise *F*_ST_ Values Pairwise *F*_ST_ values are indicated by the height of each peak. The significance of the *F*_ST_ values relative to an empirical distribution of 2,540 autosomal SNPs and 207 small indels is indicated by the peak color. Yellow, top 10%; orange, top 5%; red, top 1%. Populations are listed in the same order as in Table S3.
(2.7 MB TIF)

Figure S3 Haplotype Network Haplotypes are numbered as in Figure 4. Yellow, haplotypes carrying the insertion allele; black, haplotypes carrying the deletion allele.
(4.1 MB TIF)

Figure S4 EHH in YRI and CEU Populations EHH and REHH values are shown for the Yoruba (A and B) and European (C and D) populations. Distances are plotted based on the given SNP coordinates without accounting for the reduced size of the deletion haplotype. Solid line, insertion allele; dotted line, deletion allele.
(1.4 MB TIF)

Table S1 Individual Genotypes from HGDP (131 KB XLS)

Table S2 Deletion Frequencies for Continental Groupings (30 KB DOC)

Table S3 Frequencies for Populations from the HGDP (87 KB DOC)

Table S4 Individual Genotypes from HapMap (36 KB XLS)

Table S5 Frequencies for Each HapMap Population (27 KB DOC)

Table S6 HapMap Phase II SNPs Used to Construct Haplotype Networks (34 KB DOC)

Table S7 Definition of 49 Haplotypes Observed Over This Region (51 KB DOC)

### Accession Numbers

The GenBank (http://www.ncbi.nlm.nih.gov/Genbank) accession number for the fosmid sequence is AC192820. The sequences of *APOBEC3A* (NM_145699.2) and *APOBEC3B* (NM_004900.3) were obtained from GenBank.

## Abbreviations

CEPH: Centre d'Etude du Polymorphisme Humain
CEU: CEPH project in Utah
CHB: individuals from Han Chinese population in Beijing, China
EHH: extended haplotype homozygosity
EHL: extended haplotype length
HGDP: Human Genome Diversity Panel
JPT: individuals of Japanese ancestry from Tokyo, Japan
LD: linkage disequilibrium
REHH: relative extended haplotype homozygosity
SNP: single nucleotide polymorphism
YRI: individuals from the Yoruba population of the Ibidan Peninsula, Nigera
