# Supplementary material for: Population Stratification of a Common APOBEC Gene Deletion Polymorphism
Source: PLoS Genet. 2007 Apr 20;3(4):e63. doi: 10.1371/journal.pgen.0030063 (PMC1853121; doi:10.1371/journal.pgen.0030063)
Supplement: Supplementary file 1 [file pgen.0030063.st002.doc]

Supplementary Table 2: Frequency of *APOBEC3B* deletion grouped by broad geographic location

|  | Allele Counts | |  |
| --- | --- | --- | --- |
| Geographic Region | Deletion | Insertion | Deletion Frequency |
| Oceania | 65 | 5 | 0.9286 |
| East Asia | 174 | 298 | 0.3686 |
| Central South Asia | 55 | 333 | 0.1418 |
| Middle East | 26 | 314 | 0.0765 |
| Europe | 20 | 288 | 0.0649 |
| Africa | 2 | 220 | 0.0090 |
| America | 102 | 112 | 0.4766 |
|  |  |  |  |
| Total | 444 | 1570 | 0.2205 |
