# Supplementary material for: Population Stratification of a Common APOBEC Gene Deletion Polymorphism
Source: PLoS Genet. 2007 Apr 20;3(4):e63. doi: 10.1371/journal.pgen.0030063 (PMC1853121; doi:10.1371/journal.pgen.0030063)
Supplement: Supplementary file 2 [file pgen.0030063.st003.doc]

Supplementary Table 3: HGDP Genotype Frequencies

|  |  | Genotype Counts | | |  |
| --- | --- | --- | --- | --- | --- |
| Population | Geographic Location | D/D | D/I | I/I | HWE^a^ |
| Melanesian | Bougainville | 16 | 2 | 1 | 0.160 |
| Papuan | New Guinea | 15 | 1 | 0 | 1.000 |
| Cambodian | Cambodia | 2 | 8 | 1 | 0.257 |
| Dai | China | 3 | 7 | 0 | 0.220 |
| Daur | China | 2 | 5 | 3 | 1.000 |
| Han | China | 0 | 28 | 16 | 0.002 |
| Hezhen | China | 0 | 2 | 7 | 1.000 |
| Japanese | Japan | 1 | 12 | 15 | 0.646 |
| Lahu | China | 0 | 6 | 4 | 0.480 |
| Miao | China | 1 | 6 | 3 | 1.000 |
| Mongola | China | 0 | 8 | 2 | 0.173 |
| Naxi | China | 2 | 4 | 4 | 0.573 |
| Oroqen | China | 0 | 6 | 4 | 0.480 |
| She | China | 2 | 7 | 1 | 0.520 |
| Tu | China | 1 | 6 | 3 | 1.000 |
| Tujia | China | 2 | 4 | 4 | 0.573 |
| Xibo | China | 2 | 2 | 5 | 0.167 |
| Yakut | Siberia | 2 | 13 | 10 | 0.663 |
| Yi | China | 1 | 8 | 1 | 0.200 |
| Balochi | Pakistan | 0 | 8 | 16 | 1.000 |
| Brahui | Pakistan | 0 | 5 | 20 | 1.000 |
| Burusho | Pakistan | 1 | 9 | 15 | 1.000 |
| Hazara | Pakistan | 0 | 8 | 14 | 1.000 |
| Kalash | Pakistan | 0 | 3 | 21 | 1.000 |
| Makrani | Pakistan | 0 | 5 | 15 | 1.000 |
| Pathan | Pakistan | 2 | 6 | 13 | 0.540 |
| Sindhi | Pakistan | 0 | 4 | 19 | 1.000 |
| Uygur | China | 0 | 1 | 9 | 1.000 |
| Bedouin | Israel-Negev | 0 | 5 | 42 | 1.000 |
| Druze | Israel-Carmel | 1 | 5 | 39 | 0.223 |
| Mozabite | Algeria-Mzab | 0 | 2 | 27 | 1.000 |
| Palestinian | Israel-Central | 1 | 10 | 38 | 0.535 |
| Adygei | Russia-Caucasus | 0 | 4 | 13 | 1.000 |
| Basque | France | 0 | 3 | 21 | 1.000 |
| French | France | 0 | 1 | 23 | 1.000 |
| Italian | Italy-Bergamo | 1 | 0 | 12 | 0.040 |
| Orcadian | Orkney Islands | 0 | 3 | 13 | 1.000 |
| Russian | Russia | 0 | 6 | 19 | 1.000 |
| Sardinian | Italy | 0 | 1 | 27 | 1.000 |
| Tuscan | Italy | 0 | 0 | 7 | - |
| Bantu Kenya | Kenya | 0 | 0 | 12 | - |
| Biaka Pygmy | Central African Republic | 0 | 0 | 31 | - |
| Mandenka | Senegal | 0 | 2 | 21 | 1.000 |
| Mbuti Pygmy | Congo | 0 | 0 | 15 | - |
| San | Namibia | 0 | 0 | 6 | - |
| Yoruba | Nigeria | 0 | 0 | 24 | - |
| Colombian | Colombia | 2 | 8 | 3 | 0.602 |
| Karitiana | Brazil | 8 | 11 | 5 | 0.698 |
| Maya | Mexico | 1 | 9 | 15 | 1.000 |
| Pima | Mexico | 6 | 7 | 11 | 0.083 |
| Surui | Brazil | 13 | 7 | 1 | 1.000 |

The number of individuals genotyped as homozygous for the deletion (D/D), hemizygous (I/D), and homozygous for the insertion (I/I) are given. Homozygous deletion status was confirmed by two independent breakpoint assays for the insertion allele.

^a^Probability of Hardy-Weinberg equilibrium
