# Supplementary material for: Population Stratification of a Common APOBEC Gene Deletion Polymorphism
Source: PLoS Genet. 2007 Apr 20;3(4):e63. doi: 10.1371/journal.pgen.0030063 (PMC1853121; doi:10.1371/journal.pgen.0030063)
Supplement: Supplementary file 3 [file pgen.0030063.st005.doc]

_Supplementary Table 5: HapMap Genotype Frequencies._

|  | Genotype Counts | | |  |
| --- | --- | --- | --- | --- |
| Population | D/D | D/I | I/I | HWE |
| YRI | 0 | 3 | 57 | 1 |
| CEU | 0 | 8 | 52 | 1 |
| CHB | 7 | 19 | 19 | 0.5323 |
| JPT | 7 | 21 | 17 | 1 |
| JPT+CHB | 14 | 40 | 36 | 0.6544 |

Columns as indicated in Supplementary table 4.
