# Supplementary material for: Population Stratification of a Common APOBEC Gene Deletion Polymorphism
Source: PLoS Genet. 2007 Apr 20;3(4):e63. doi: 10.1371/journal.pgen.0030063 (PMC1853121; doi:10.1371/journal.pgen.0030063)
Supplement: Supplementary file 4 [file pgen.0030063.st006.doc]

Supplemental Table 6:

HapMap Phase II SNPs used in haplotype network analysis.

| BD35 Coordinate | SNP ID |
| --- | --- |
| 37673148 | rs5750716 |
| 37673559 | rs5757401 |
| 37673846 | rs6001337 |
| 37674117 | rs5995640 |
| 37674276 | rs6001338 |
| 37674506 | rs6001339 |
| 37674722 | rs17370615 |
| 37675450 | rs5995641 |
| 37675537 | rs5995642 |
| 37675966 | rs17298479 |
| 37676118 | rs9611054 |
| 37677672 | rs12157810 |
| 37713920 | rs2019907 |
| 37716133 | rs6001361 |
| 37716696 | rs738308 |
| 37716750 | rs11089916 |
| 37716796 | rs2142833 |
| 37716980 | rs6001363 |
| 37718597 | rs8140586 |
| 37720818 | rs10470278 |
| 37721871 | rs9611073 |
