# Supplementary material for: Population Stratification of a Common APOBEC Gene Deletion Polymorphism
Source: PLoS Genet. 2007 Apr 20;3(4):e63. doi: 10.1371/journal.pgen.0030063 (PMC1853121; doi:10.1371/journal.pgen.0030063)
Supplement: Supplementary file 5 [file pgen.0030063.st007.doc]

Supplemental Table 7: Observed Haplotypes

I/D indicates presence of insertion or deletion allele. SNP positions as given in Supplemental Table 6

| Haplotype Number | Sequence |
| --- | --- |
| 1 | CTAGTCGCCGGCIGCTAGCGC |
| 2 | CTAGTCGCCGGCIGCTGGCGC |
| 3 | CTAGTCGCCGGCIGTTGGCGC |
| 4 | CTAGTCGTTGGAIACTGATGC |
| 5 | CTAGTCGTTGGAIGCTAGCGC |
| 6 | CTAGTCGTTGGAIGCTGGCGC |
| 7 | CTGACCGCCGGAIACTGATGC |
| 8 | CTGACCGCCGGAIGCTGGCGC |
| 9 | CTGACCGCCGGCIGCTGGCGC |
| 10 | CTGACCGTTGGAIACTGATGC |
| 11 | CTGACCGTTGGAIGCTAGCGC |
| 12 | CTGACCGTTGGAIGCTGGCGC |
| 13 | CTGACTGCCGGCIGCTGGCGC |
| 14 | CTGGCCGCCGGAIGCTAGCGC |
| 15 | CTGGCCGCCGGAIGCTGGCGC |
| 16 | CTGGTCACCGGAIACCGATGC |
| 17 | CTGGTCACCGGAIACTGATGC |
| 18 | CTGGTCACCGGAIGCTAGCGC |
| 19 | CTGGTCACCGGAIGCTGGCGC |
| 20 | CTGGTCACCGGCIACTGATGC |
| 21 | CTGGTCACCGGCIGCTGGCGC |
| 22 | CTGGTCACTGGAIGCTGGCGC |
| 23 | CTGGTCGCCAGAIACCGATGC |
| 24 | CTGGTCGCCAGAIACTGATGC |
| 25 | CTGGTCGCCAGAIACTGGCGC |
| 26 | CTGGTCGCCAGAIGCTAGCGC |
| 27 | CTGGTCGCCAGAIGCTGGCGC |
| 28 | CTGGTCGCCGGADACTGATGC |
| 29 | CTGGTCGCCGGADATTGATGC |
| 30 | CTGGTCGCCGGAIACCGATGC |
| 31 | CTGGTCGCCGGAIACTGATGC |
| 32 | CTGGTCGCCGGAIGCTAGCGC |
| 33 | CTGGTCGCCGGAIGCTGGCGC |
| 34 | CTGGTCGCCGGCDGCTGGCGC |
| 35 | CTGGTCGCCGGCIACTGATAC |
| 36 | CTGGTCGCCGGCIACTGATGC |
| 37 | CTGGTCGCCGGCIGCTAGCGC |
| 38 | CTGGTCGCCGGCIGCTGATGC |
| 39 | CTGGTCGCCGGCIGCTGGCGC |
| 40 | CTGGTCGCTGGAIACTGATGC |
| 41 | CTGGTCGCTGGAIGCTAGCGC |
| 42 | CTGGTCGCTGGAIGCTGGCGC |
| 43 | CTGGTCGCTGGCIACTGATGC |
| 44 | CTGGTCGCTGGCIGCTAGCGC |
| 45 | CTGGTCGCTGGCIGCTGGCGC |
| 46 | CTGGTCGCTGGCIGTTGGCGC |
| 47 | CTGGTCGTTGGAIACTGATGC |
| 48 | CTGGTCGTTGGAIGCTAGCGC |
| 49 | CTGGTCGTTGGAIGCTGGCGC |
